# Assessing clinical complete response after neoadjuvant systemic therapy in muscle-invasive bladder cancer: a systematic review

**DOI:** 10.1007/s00345-025-06146-7

**Published:** 2025-12-24

**Authors:** Navid Roessler, Marcin Miszczyk, Paolo Gontero, Keiichiro Miyajima, Shota Inoue, Ahmed R. Alfarhan, Abdulrahman S. Alqahtani, Markus von Deimling, Malte W. Vetterlein, Jeremy Yuen-Chun Teoh, David D’Andrea, Margit Fisch, Shahrokh F. Shariat

**Affiliations:** 1 Department of Urology, Comprehensive Cancer Center, Vienna General Hospital, Medical University of Vienna, Währinger Gürtel 18-20, Vienna, A-1090 Austria; 2 Department of Urology, University Medical Center Hamburg-Eppendorf, Hamburg, Germany; 3 Collegium Medicum, Faculty of Medicine, WSB University, Dąbrowa Górnicza, Poland; 4 Department of Urology, Città della Salute e della Scienza, University of Torino School of Medicine, Torino, Italy; 5 Department of Urology, Jikei University School of Medicine, Tokyo, Japan; 6 Department of Urology, Dentistry and Pharmaceutical Sciences, Okayama University Graduate School of Medicine, Okayama, Japan; 7 Department of Urology, Prince Saud Bin Jalawi Hospital, Al Ahsa Health Cluster, Al Ahsa, KSA Saudi Arabia; 8 Department of Urology, Second Health Cluster Riyadh, Ministry of Health, Riyadh, KSA Saudi Arabia; 9 S.H. Ho Urology Centre, Department of Surgery, The Chinese University of Hong Kong, Hong Kong, China; 10 Department of Urology, Weill Cornell Medical College, New York, NY USA; 11 Department of Urology, University of Texas Southwestern, Dallas, TX USA; 12 Department of Urology, Second Faculty of Medicine, Charles University, Prague, Czech Republic; 13 Hourani Center for Applied Scientific Research, Al-Ahliyya Amman University, Amman, Jordan; 14 Karl Landsteiner Institute of Urology and Andrology, Vienna, Austria

**Keywords:** Clinical response, Bladder cancer, Bladder preservation, Muscle-invasive, Restaging

## Abstract

**Purpose:**

Reliable assessment of clinical complete response (cCR) is essential to facilitate bladder-sparing treatment approaches in patients with muscle-invasive bladder cancer (MIBC) undergoing neoadjuvant systemic therapy, yet there is no agreement on the choice of diagnostic modalities for its assessment. We aimed to summarize strategies for assessing cCR in MIBC to facilitate development of bladder-preservation strategies.

**Methods:**

In this prospectively registered systematic review (CRD420251088678), we searched MEDLINE, Embase, and Web of Science in July 2025 for studies reporting on cCR in MIBC patients undergoing neoadjuvant therapy prior to definitive local therapy or bladder preservation strategies. Risk-of-bias (RoB) was assessed using ROBINS-I tool.

**Results:**

Out of 1696 individual records, 41 studies comprising 3588 patients were included in this systematic review. Majority of studies used cisplatin-based neoadjuvant therapies (*n* = 2941, 23 studies), with eight studies including perioperative immunotherapy (*n* = 647). For cCR evaluation, 12 studies (*n* = 1064) used only medical imaging (including CT, MRI, or FDG-PET/CT), three studies (*n* = 231) performed re-TURBT, one study (*n* = 56) used cystoscopy, and the remaining 25 studies (*n* = 2237) assessed cCR through combinations of medical imaging and clinical evaluation. Reported cCR rates ranged from 4.2% to 45% for CT, 15% to 78% for MRI, 76% for FDG-PET/CT, 25% to 46% for TURBT, 50% for cystoscopy, and 7.4% to 87% in studies combining medical imaging with local clinical evaluation. Most studies were rated as having moderate RoB concerns, while one preliminary report was classified as having serious RoB due to limited sample size.

**Conclusion:**

We found substantial heterogeneity in cCR assessment modalities, limiting comparability across bladder-sparing studies and highlighting the need for standardization. Data on patients receiving neoadjuvant chemo-immunotherapy remain scarce, and none incorporated urinary tumor DNA in cCR definitions. Serial MRI, with or without invasive assessment, appears promising for selecting candidates for bladder preservation.

**Supplementary Information:**

The online version contains supplementary material available at 10.1007/s00345-025-06146-7.

## Introduction

The current standard of care treatment for patients with non-metastatic muscle-invasive bladder cancer (MIBC) is radical cystectomy (RC) with perioperative systemic therapy, comprising chemotherapy and/or immune therapy [1, 2]. Neoadjuvant systemic therapy (NAST) prior to RC improves survival outcomes compared to RC alone [3–5], without significant increase in complications and adverse events [6, 7]. Given the high rates of pathological complete response (pCR) following NAST, and its strong association with favourable oncological outcomes, there is increasing interest in identifying surrogate endpoints that may facilitate bladder-sparing strategies and potentially avoid the need for definitive local therapy in selected patients [8, 9]. In this context, clinical complete response (cCR) after NAST represents a promising endpoint that, if accurately defined and validated, could support bladder-sparing (BSP) approaches in carefully selected patients with MIBC [10, 11]. However, the lack of standardized definitions and variability in restaging methods after NAST currently limit the reliability and clinical utility of cCR as a decision-making tool [12]. BSP strategies offer the potential for oncologic control while preserving bladder function and improving health-related quality of life compared to RC by avoiding overtreatment. To safely select patients for BSP, it is essential to develop and validate accurate, reproducible, and easily implementable methods for post-NAST response assessment.

In this systematic review, we comprehensively evaluated current restaging modalities for cCR assessment in patients with MIBC, aiming to guide bladder-sparing approaches by identifying the reliable assessments methods for the safe selection of patients for organ-preserving strategies.

## Methods

**Fig. 1:**
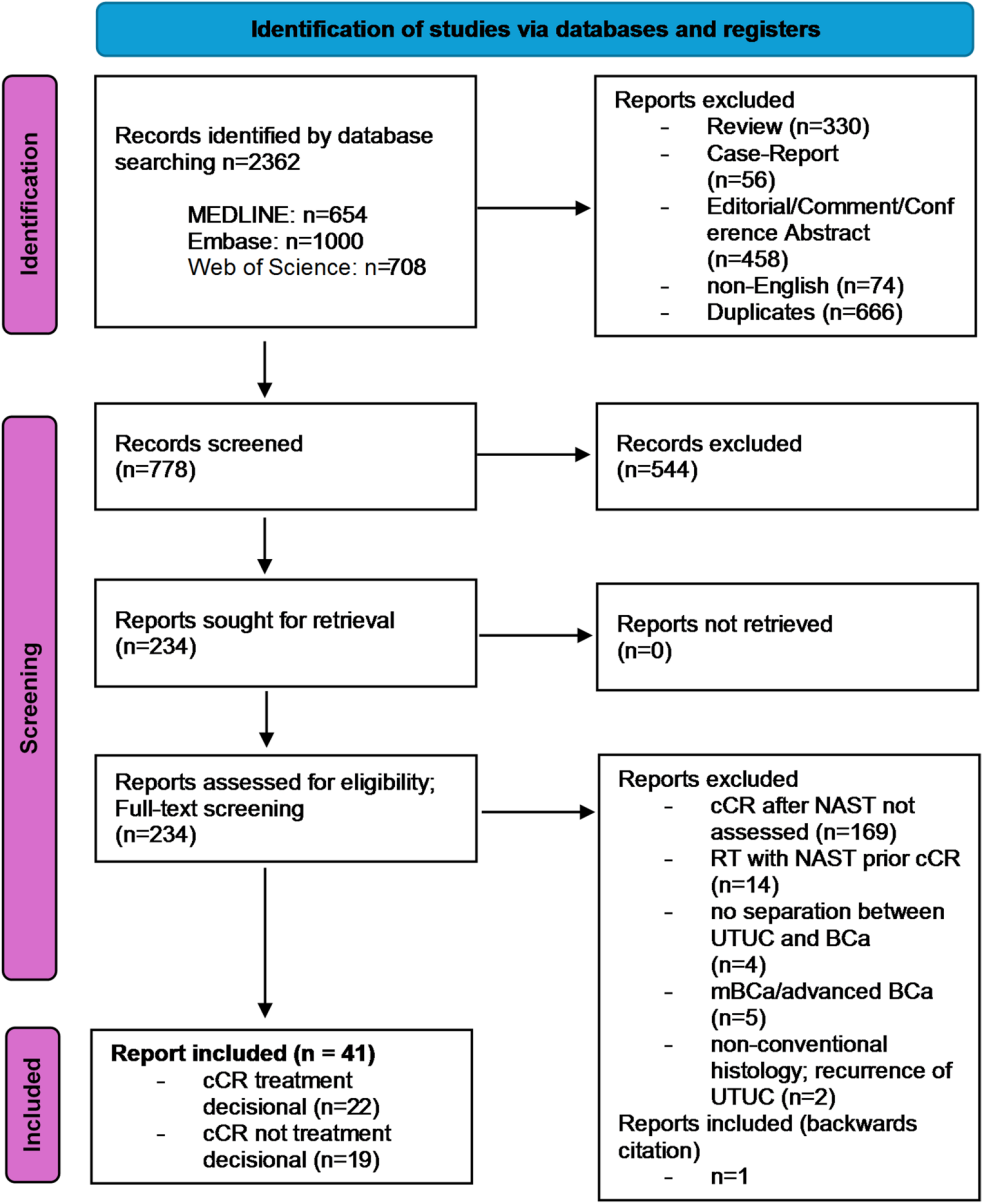
Preferred Reporting Items for Systematic Reviews and Meta-analyses (PRISMA) - flow diagram for new systematic reviews which included searches of databases and registers only

This systematic review was registered with the International Prospective Register of Systematic Reviews (CRD420251088678) and conducted in accordance with the Preferred Reporting Items for Systematic Reviews and Meta-analyses (PRISMA) flowchart (Fig. 1) and the AMSTAR2 checklist (Supplementary File 5) [13, 14]. Ethical approval and consent to participate were not applicable; no specific funding was received for this work.

### Study selection

The research question and inclusion criteria were defined using the Population (P), Exposure (E), Outcome (O), and study design (S) framework (Supplementary File 4). We searched MEDLINE (via PubMed), Embase and Web of Science Core Collection for studies evaluating patients with MIBC who underwent clinical restaging to assess cCR (cCR; defined as cT0, with or without nodal assessment [cT0N0 if N status was evaluated]; no evidence of residual disease) after receiving at least one cycle of neoadjuvant systemic therapy, prior to definitive local therapy or bladder preservation surveillance strategies. We included all retrospective and prospective reports, including subset and post-hoc analyses, that reported cCR rates among patients with MIBC. To maintain cohort homogeneity, studies including concurrent radiotherapy with neoadjuvant systemic therapy and metastatic bladder cancer were excluded. Non-English language manuscripts, studies not providing original data, editorials, case-reports, and review articles were excluded.

The search strategy was performed in July 2025, and the detailed search strategy is provided in Supplementary File 2. Reports were merged and de-duplicated using EndNoteX9 (Clarivate) and the title-abstract screening was conducted independently by two authors. Following title-abstract screening, full-text reports were retrieved and screened for relevance independently. Backward citation searching was performed to identify potentially relevant additional records. At each step of the review, conflicts were resolved through consensus among co-authors.

### Data extraction

Two authors independently extracted data, including the first author’s name, year of publication, patient characteristics (e.g., age, sample size, neoadjuvant therapy regimen), restaging details (e.g., type of restaging modality [cystoscopy, urine cytology, TransUrethral-Resection of the Bladder Tumor (TURBT), Computed Tomography (CT), Magnetic Resonance Imaging (MRI), Fluorodeoxyglucose Positron Emission Tomography/CT (FDG PET/CT)]), and rates of cCR and clinical partial response (cPR; defined as significant but incomplete reduction in tumor size or extent). All conflicts that arose during the data extraction process were resolved through discussion between authors.

### Risk of bias assessment

Each study was evaluated independently by two authors using the Risk Of Bias in Non-randomized Studies-of Interventions (ROBINS-I) tool for non-randomized studies [15].

## Results

Based on our inclusion criteria, 41 studies comprising 3588 patients were included for this systematic review, as presented in Table 1[16–56]. Among these, 12 studies (*n*= 926) were prospective in design [16, 17, 25, 28, 31, 35, 38, 42, 44, 47, 48, 56], and the remaining 29 studies (*n* = 2662) were retrospective. In 19 studies (*n*= 1825) [16–34], cCR did not influence treatment decisions, while in 22 studies (*n*= 1763), treatment was guided based on cCR status after NAST [35–56]. The clinical stage at diagnosis prior to neoadjuvant therapy was predominantly T2. Cisplatin-based neoadjuvant therapies were administered in 23 studies (*n* = 2941), with eight studies including perioperative immunotherapy (*n*= 647) [22, 26, 28, 31, 46–48, 53]. Most patients received three to four treatment cycles.

Twelve studies (*n* = 1064) used medical imaging alone to evaluate cCR, including CT (*n*= 673, six studies) [18–23], MRI (*n*= 346, five studies) [25–29], or FDG-PET/CT (*n*= 45, one study) [24]. Three studies (*n*= 231) performed TURBT for restaging to assess cCR [17, 35, 36], and in one study (*n*= 56) cystoscopy was performed prior to surgery [16]. The remaining 25 studies (*n* = 2237) assessed cCR through combination of clinical modalities, including imaging (CT or MRI), cystoscopy with or without biopsy, urine cytology, and/or TURBT. Reported cCR rates ranged from 25% to 46% for TURBT alone and reached 50% for cystoscopy without biopsy. CT-based assessments demonstrated cCR rates between 4.2% and 45%, while MRI assessments ranged from 15% to 78%. FDG-PET/CT yielded a cCR rate of 76%. Studies employing combinations of clinical modalities reported cCR rates from 7.4% to 87%.

**Table 1:** Demographics and clinical characteristics of the included studies

Study	No. of patients	Age, years	Sex	cT stage pre-NAST	NAST regime	No. of NAST cycles	cCR assessment modality	cCR, *n* (%)	cPR, *n* (%)
Treatment Decisions after NAST Independent of cCR Assessment									
Asad, D., et al. [16]	56	mean 67 (range 39–80)	42 (75) M; 14 (25) F	cT2: 43 (77); cT3: 12 (21); cT4a: 1 (2)	ddMVAC: 48 (86); MVAC: 2 (4); Gem/Cis: 5 (9); Gem/Carbo 1 (2)	2: 6 (11); 3: 45 (80); 4: 4 (7); 6:1 (2)	cystoscopy	28 (50)	NR
Mehr, J. P., et al. [17]	53	median 69 (range 61–75)	43 (81) M; 10 (9) F	NR	NR	NR	TURBT	13 (25)	16 (30)
Abdelrahman, I., et al. [18]	72	median 61 (34–87)	62 (86) M; 10 (14) F	cTx: 1 (1.4); cT2: 27 (38); cT3b 38 (53); cT4: 6 (8.3)	Gem/Cis: 69 (96); Gem: 1 (1); Carbo: 2 (3)	median 1 (1–8)	CT	3 (4.2)	44 (61)
Fukui, T., et al. [19]	59	median 68 (range 50–81)	NR	cT2: 24 (41); cT3: 32 (54); cT4: 3 (5.1)	MVAC: 18 (31); MEC: 3 (5.1); Gem/Cis: 26 (44); Gem/CBDCA: 11 (19); EP: 1 (1.7)	NR	CT	9 (15)	36 (61)
Mertens, L. S., et al. [20]	167	Mean 59 (SD ± 8.8)	128 (77) M; 39 (23) F	cT2: 59 (35); cT3: 48 (29); cT4: 60 (36)	MVAC: 112 (67); Gem/Cis: 14 (8.3); Gem/Carbo: 41 (25)	4: 124 (74)	CT	40 (32)	72 (58)
Mogos, H., et al. [21]	80	median 68 (range 44–80)	60 (75) M; 20 (25) F	cT2: 31 (39; cT3: 38 (47); cT4a: 11 (14)	MVAC/ddMVAC: 75 (94); Gem/Cis: 5 (6)	1: 1 (1); 2: 9 (11); 3: 46 (58); 4: 23 (29); 5: 1 (1)	CT	36 (45)	NR
Zhang, H., et al. [22]	154	NR	126 (82) M; 28 (18) F	cT2: 78 (51); cT3: 46 (30); cT4: 30 (19)	Gem/Cis: 77 (50); Gem/Cis + immune checkpoint inhibitor: 77 (50)	≥ 3: 154 (100)	CT	39 (25)	57 (37)
Alam, S. M., et al. [23]	141	median 65	109 (77) M; 32 (33) F	NR	Gem/Cis: 79 (56); MVAC: 37 (26)	NR	CT	59 (42)	52 (47)
Fitoussi, O., et al. [24]	45	mean 66 (SD ± 6)	39 (87) M; 6 (13) F	pTa: 2 (4); pT2: 43 (96)	ddMVAC: 26 (58); Gem/Cis or Gem/Carbo: 19 (42)	ddMVAC: 6; Gem/Cis: 3	FDG-PET/CT	34 (76)	9 (20)
Elkarta, A., et al. [25]	104	mean 63 (SD ± 6.7)	95 (91) M; 9 (8.7) F	cT2b: 43 (41); cT3b: 48 (46); cT4a: 13 (13)	Gem/Cis: 104 (100)	mean 4.6 (SD 0.9)	MRI	39 (38)	NR
Brembilla, G., et al. [26]	110	median 67 (IQR 61–74)	96 (87) M; 14 (13) F	cT2: 58 (53); cT3-4: 52 (47)	Pembrolizumab (PURE-01)	3: 110 (100)	mpMRI	86 (78)	NR
De Maeseneer, D., et al. [27]	40	NR	32 (80) M; 8 (20) F	NR	ddMVAC: 40 (100)	1: 1(2.5); 2: 2 (5); 3: 2 (5); 4: 35 (88)	mpMRI	6 (15)	NR
Necchi, A., et al. [28]	82	NR	NR	NR	Pembrolizumab (PURE-01)	3: 110 (100)	mpMRI	37 (47)	NR
Pecoraro, M., et al. [29]	10	NR	6 (60) M; 4 (40) F	NR	Gem/Cis: 9 (90); CMV: 1 (10)	mean 3	mpMRI	3 (30)	NR
Becker, R. E. N., et al. [30]	318	NR	261 (82) M; 57 (18) F	NR	Gem/Cis: 222 (70); Gem/Carbo: 19 (6); MVAC: 11(3.5); ddMVAC: 20 (6.3); other: 46 (14.5)	NR	CT, cystoscopy with or without TURBT, bimanual examination	53 (47)	NR
Kim, H., et al. [31]	51	median 66 (range 48–84)	43 (84) M; 8 (16) F	cT2: 33 (65); cT3:13 (26); cT4a: 5 (10)	Gem/Cis + Nivolumab: 51 (100)	3–4: 51 (100)	CT or cystoscopy, urine cytology	30 (59)	
Reese, A. C., et al. [32]	62	mean 64	50 (81) M; 12 (19) F	NR	Gem/Cis: 47 (76); Gem/Carbo: 3 (5); other: 12 (19)	≥ 3: 62 (100)	CT/MRI, cystoscopy with or without biopsy, bimanual examination	22 (35)	6 (9.7)
Piao, C., et al. [33]	161	mean 65 (SD ± 8.7)	126 (78) M; 35 (22) F	cT2: 138 (86); cT3: 19 (12); cT4: 4 (2.5)	Gem/Cis: 161 (100)	3–4: 161 (100)	CT, cystoscopy with biopsy or TURBT, urine cytology	161 (100)	NR
Scattoni, V., et al. [34]	60	mean 56 (range 38–75)	49 (82) M; 11 (18) F	cT2: 2 (3.3); cT3a: 20 (33); cT3b: 29 (48); cT4: 9 (15)	CMV: 60 (100)	≥ 2: 60 (100)	CT/MRI, cystoscopy, urine cytology	6 (10)	28 (47)
Treatment Decision after NAST Dependent of cCR Assessment									
deVere White, R. W., et al. [35]	74	median 69 (range 49–83)	56 (76) M; 18 (24) F	cT2: 52 (70); cT3: 17 (23); cT4a: 5 (7)	Gem/Carbo/Paclitaxel: 74 (100)	3: 74 (100)	TURBT	34 (46)	NR
Sternberg, C. N., et al. [36]	104	median 65 (range 30–82)	96 (92) M; 8 (8) F	cT2: 37 (36); cT3a: 33 (32); cT3b: 26 (25); cT4: 8 (7.7)	MVAC: 104 (100)	3: 104 (100)	TURBT	48 (46)	NR
Herr, H. W., et al. [37]	111	NR	NR	NR	MVAC: 111 (100)	4: 111 (100)	cystoscopy with biopsy, urine cytology	60 (54)	NR
Splinter, T. A., et al. [38]	149	median 64 (range 39–76)	131 (88) M; 18 (12) F	cT3a: 78 (52); cT3b-T4: 71 (48)	CMV: 75 (100)	NR	cystoscopy with biopsy, bimanual examination	11 (7.4)	66 (44)
Sternberg, C. N., et al. [39]	47	median 64 (range 47–82)	45 (96) M; 2 (4) F	cT2: 13 (28); T3a: 14 (30); T3b: 19 (40); T4a: 1 (2)	MVAC: 47 (100)	3: 47 (100)	CT, TURBT, urine cytology	19 (41)	17 (37)
Onishi, T., et al. [40]	71	median 72 (range 49–90)	44 (76) M; 14 (24) F	cT2: 42 (72); cT3: 14 (26); cT4: 1 (1.7)	Gem/Cis: 54 (93); MVAC: 2 (3.4); Gem/Carbo: 2 (3.4)	median 3 (range 2–5)	CT, TURBT	33 (46)	NR
Yap, S. A., et al. [41]	60	median 71 (range 41–91)	49 (82) M; 11 (18) F	NR	NR	3–4: 60 (100)	CT, TURBT	32 (53)	NR
Agrawal, C., et al. [42]	30	median 61	NR	cT2b: 18 (60)	Gem/Cis: 30 (100)	1: 1 (3.3); 2: 3 (10); 3: 26 (87)	CT, cystoscopy	17 (57)	8 (27)
Herr, H. W. [43]	63	median 62 (range 32–79)	47 (75) M; 16 (25) F	cT2: 36 (57); cT3-T4: 27 (43)	MVAC: 51 (81); other: 12 (19)	NR	CT, cystoscopy with biopsy	63 (100)	NR
Dracham, C. B., et al. [44]	40	median 62 (range 50–74)	40 (100) M	cT2: 18 (45); cT3: 16 (40); cT4a: 6 (15)	Gem/Cis: 40 (100)	3: 40 (100)	CT, cystoscopy with or without biopsy	9 (23)	26 (65)
Meyer, A., et al. [45]	32	mean 68 (SD ± 9.6)	26 (81) M; 6 (19) F	NR	MVAC: 21 (66); Gem/Cis: 8 (25); Gem/Carbo: 2 (6.3); Carbo: 1 (3.1)	NR	CT, cystoscopy with biopsy, urine cytology	32 (29)	NR
Chang, P. H., et al. [46]	53	median 71 (range 41–91)	41 (77) M; 12 (23) F	cT2: 27 (51); cT3: 26 (49)	Gem/Cis: 23 (43); Pembrolizumab: 23 (43); Gem/Carbo: 7 (13)	≥ 3: 53 (100)	CT/MRI, cystoscopy with or without biopsy, urine cytology	23 (43)	NR
Cho, S. W., et al. [47]	76	median 67 (range 40–85)	63 (83) M; 13 (17) F	cT2: 66 (87); cT3-cT4a: 10 (13)	Gem/Cis: 64 (84); Gem/Cis + Nivolumab: 12 (16)	NR	CT/MRI or cystoscopy with urine cytology	66 (87)	NR
Galsky, M. D., et al. [48]	76	median 69 (range 39–85)	60 (79) M; 16 (21) F	cT2: 43 (57); cT3: 24 (32); cT4: 9 (12)	Gem/Cis + Nivolumab: 76 (100)	4: 76 (100)	CT/MRI, cystoscopy with or without biopsy, urine cytology	33 (43)	NR
Hafeez, S., et al. [49]	94	median 65 (range 34–83)	82 (87) M; 12 (13) F	cT2: 70 (74); cT3: 18 (19); cT4: 6 (6.3)	Gem/Cis: 43 (46); ddMVAC: 39 (41); Gem/Carbo: 12 (13)	median 3 (range 1–5)	cystoscopy with or without biopsy, with or without CT/MRI	53 (60)	25 (28)
Han,B., et al. [50]	127	median 69 (range 36–100)	103 (81) M; 24 (19) F	cT2: 74 (58); cT3-T4: 53 (42)	Cis/EFC: 127 (100)	NR	CT/MRI, cystoscopy with biopsy, urine cytology	91 (72)	NR
Hatcher, P. A., et al. [51]	39	median 61 (range 36–77)	33 (85) M; 6 (15) F	NR	MVAC: 19 (49); Cis/Eto: 15 (38); CMV: 5 (13)	median 2.8 (range 1–6)	CT, cystoscopy with biopsy, urine cytology	12 (31)	23 (59)
Liang, S., et al. [52]	46	median 68 (range 46–84)	40 (87) M; 6 (13) F	cT2: 30 (65); cT3: 16 (35)	Cis/Epi/5-FU or Cis/Epi/Camptothecin: 46 (100)	3: 46 (100)	CT/MRI, cystoscopy with biopsy, urine cytology	32 (70)	NR
Luo, C., et al. [53]	45	Median 64 (IQR 59–71)	37 (82) M; 8 (18) F	cT2: 26 (58); cT3: 11 (24); cT4a: 8 (18)	Cis/Gem/Tislelizumab: 45 (100)	4: 45 (100)	MRI, cystoscopy with biopsy, urine cytology	29 (64)	NR
Mazza, P., et al. [54]	148	median 62 (range 32–88)	119 (80) M; 29 (20) F	cT2: 146 (99); cT3: 2 (1)	Gem/Cis: 93 (63); MVAC: 46 (31); Other: 9 (6)	≥ 4: 110 (81); <4: 26 (19)	CT, cystoscopy with biopsy, urine cytology	148 (100)	NR
Mofid, B., et al. [55]	140	mean 66 (SD ± 10)	130 (93) M; 10 (7) F	cT2: 80 (57); cT3: 48 (34); T4a: 11 (7.9)	Gem/Cis: 79 (56); Gem/Carbo: 61 (44)	NR	CT, cystoscopy with biopsy, urine cytology	50 (38)	NR
Sung, H. H., et al. [56]	138	median 68 (range 40–86)	119 (86) M; 19 (14) F	cT2: 71 (51); cT3: 52 (38); cT4a: 15 (11)	Gem/Cis: 138 (100)	median 3 (range 1–4)	CT or cystoscopy with urine cytology	54 (39)	NR

*cCR* clinical complete response, *cPR* clinical partial response, *CT* computed tomography, *FDG-PET/CT* fluorodeoxyglucose positron emission tomography/computed tomography, *F* female, *M* male, *MRI* magnetic resonance imaging, *mpMRI* multiparametric magnetic resonance imaging, *NAST* neoadjuvant systemic therapy, *NR* not reported, *TURBT* transurethral resection of bladder tumor

### Assessment of risk of bias

The risk of bias judgments of each domain for each included study are summarised in Supplementary File 3. According to the ROBINS-I tool for non-randomized studies, 12 studies were rated as having a low risk of bias [26, 28, 30, 31, 37, 40, 42, 44, 47, 48, 53, 56]. The remaining studies were assessed as having a moderate risk of bias, primarily due to the use of only a single restaging modality to assess cCR and their retrospective study design. One study, being a preliminary report, was rated as having a serious risk of bias due to a small patient cohort (*n*= 10) and the use of only a single restaging modality [29].

### Treatment decisions independent of cCR assessment

In 19 studies (*n* = 1825), post-NAST treatment for MIBC was guided irrespective of clinical response, with therapeutic decisions made independently of cCR assessment.

#### cCR assessment based on cystoscopy and TURBT alone

Asad et al. prospectively evaluated visual cCR via cystoscopy immediately prior to RC in 56 patients. Every second patient (50%, *n*= 28) with a visual cCR assessment had false negative cCR findings, including six with residual muscle-invasive disease [16]. Mehr et al. retrospectively analyzed restaging TURBT prior to RC in 53 patients and found that despite a 25% (*n* = 13) cCR rate, 35% (*n*= 29) were upstaged at RC [17]. These findings underscore the limitations of visual inspection and TURBT in reliably assessing cCR.

#### cCR assessment based on CT and FDG-PET/CT

Six studies using CT alone reported cCR rates post-NAST ranging from 4.2% to 45%. Abdelrahman et al. reported a 4.2% (*n* = 3) cCR rate, yet the corresponding pCR rate at RC was 15% (*n*= 11) [18]. Similarly Alam et al. reported a positive predictive value of cCR of approximately 50% for pCR at RC [23].Mogos et al. reported sensitivity of 64% and specificity of 36% of cCR for the detection of pCR [21]. Fukui et al. reported cCR rates of 15% (*n*= 9) [19]. [  Mertens et al. and Zhang et al. reported cCR rates of 32% (*n* = 40) and 25% (*n*= 39), respectively [20, 22]. Zhang et al. demonstrated a significantly higher cCR rate in patients receiving neoadjuvant immunotherapy combined with chemotherapy compared to chemotherapy alone (*p* < 0.001). Overall, these results highlight the limited ability of CT to reliably detect cCR. One study by Fitoussi, O., et al. , which assessed FDG-PET/CT after NAST, showed a cCR rate of 76% (*n*= 34), with high sensitivity (95%) and negative predictive value (91%) for predicting pCR at RC, but limited specificity (42%) and positive predictive value (59%) [24].

#### cCR assessment based on MRI

Five studies reported MRI-based cCR rates ranging from 15% to 78%. Two studies from the PURE-01 trial demonstrated mpMRI’s high accuracy and reproducibility for predicting pCR at RC: Brembilla, G., et al reported sensitivities up to 98% for detecting residual muscle-invasive disease, while Necchi, A., et al. found significant associations between mpMRI-based cCR and pCR (*p*< 0.001) [26, 28]. De Maeseneer, D., et al. found high specificity (96%) but lower sensitivity (36%) of mpMRI-assessed cCR in detecting pCR [27]. Elkarta, A., et al. reported a 38% (*n*= 39) cCR rate via MRI [25]. Pecoraro, M., et al. found that three patients with cCR out of ten patients treated with NAST also had pCR at RC [29]. Overall cCR evaluated via MRI demonstrated high diagnostic accuracy, particularly for detecting residual disease.

#### cCR assessment based on imaging and local clinical evaluation

Five studies reported multimodal post-NAST cCR rates ranging from 10% to 59%. In a prospective phase II trial , patients receiving neoadjuvant nivolumab plus gemcitabine/cisplatin achieved a 59% (*n* = 30) cCR rate, assessed by CT or cystoscopy with urine cytology. This cCR was significantly associated with pCR (*p*= 0.037) at RC in applicable patients, although 12 cCR patients (24%) declined RC and received chemoradiotherapy [31]. Becker, R. E. N., et al. evaluated re-TURBT with CT in 114 patients and demonstrated that clinical reassessment alone falsely downstaged 32% (*n*= 37) compared to final pathological staging at RC [30]. Piao et al. retrospectively analysed 161 patients with cCR after NAST, assessed by CT, cystoscopy with biopsy/TURBT, and urine cytology, and demonstrated that 64% (*n*= 103) of patients achieved pCR at RC [33]. Reese, A. C., et al. showed that restaging with CT/MRI and cystoscopy with or without biopsy altered treatment decisions in 9.7% (*n*= 6) and demonstrated superior concordance with pathological staging at RC versus initial clinical staging (κ = 0.17 vs. κ = 0.02) [32]. Scattoni, V., et al. reported a cCR rate of 10% (*n* = 6), assessed by CT/MRI, cystoscopy, and urine cytology, with 8.3% (*n* = 5) achieving pCR at RC and 48% (*n*= 29) experiencing tumor downstaging. Despite these findings, the modest cCR rate underscores challenges in accurately defining clinical remission in this context [34].

### Treatment decisions dependent of cCR assessment

In 22 studies (*n* = 1763), treatment decisions following NAST for MIBC were based on restaging assessment. Two studies (*n* = 178) utilized re-TURBT as the sole modality for cCR evaluation, whereas the remaining 20 studies (*n* = 1585) employed a multimodal restaging approach combining imaging, endoscopy, and/or biopsy/TURBT.

#### cCR assessment based on cystoscopy and TURBT alone

In a phase II trial by deVere White et al. 46% (*n*= 34) of patients achieved cCR at re-TURBT and were given the option of surveillance or immediate RC. Six of ten patients with cCR (60%) who underwent immediate RC were found to have residual disease [35]. Similarly  Sternberg et al. reported a 46% cCR rate (*n* = 48) at re-TURBT. Of these, 44% (*n* = 23) managed with TURBT alone and 31% (*n*= 4) undergoing partial cystectomy remained alive with an intact bladder at long-term follow-up (median 56 and 88 months, respectively) [36].

#### cCR assessment based on imaging and local clinical evaluation

Galsky et al. reported a cCR rate of 43% (*n*= 33), assessed by CT/MRI, cystoscopy with or without biopsy, and urine cytology, with 97% of these patients opting to defer immediate cystectomy; at a median follow-up of 30 months, eight patients eventually underwent cystectomy for local recurrence, including one with an abnormal MRI but no malignancy on TURBT or final pathology [48]. Similarly [ Luo et al. found a cCR rate of 64% (*n*= 29), assessed by MRI, cystoscopy with biopsy, and urine cytology; patients achieving cCR showing 96% bladder-intact disease-free survival at 24 months, supporting cCR as a surrogate for favourable outcomes [53].

Long-term outcomes after bladder preservation in cCR patients have been encouraging. Mazza et al. reported a 5-year cystectomy-free survival of 76% among 148 cCR patients, assessed by CT, cystoscopy with biopsy, and urine cytology. Salvage cystectomy was performed in 75% (*n*= 9) of muscle-invasive relapses, with a 5-year disease-specific survival of 90% [54]. Similarly Herr et al. [] reported 10-year follow-up data showing that 74% (*n* = 32) of cCR patients, assessed by cystoscopy with biopsy and urine cytology, and managed with bladder-sparing surgery (TURBT, partial cystectomy) were alive, with 58% (*n*= 25) retaining an intact bladder [37].

The prognostic value of response to NAST in selecting patients for bladder preservation radiotherapy was highlighted by Hafeez et al.   reporting 82% bladder preservation among patients undergoing radiotherapy after NAST, with cCR (*n*= 53) assessed by cystoscopy with or without biopsy, and with or without CT/MRI [49]. Sung et al. evaluated neoadjuvant chemotherapy followed by selective bladder preservation chemoradiotherapy in 54 patients with cCR, assessed by CT or cystoscopy with urine cytology, and reported a 90% bladder preservation rate after a median follow-up of 34 months [56].

In addition to systemic and radiotherapy-based approaches intra-arterial chemotherapy has also demonstrated favourable outcomes in achieving cCR and supporting bladder preservation. 52Liang et al. reported a 70% cCR rate and over 80% bladder preservation at nearly three years of follow-up in 32 patients while Han et al. documented a 72% cCR rate in 91 patients; both studies assessed cCR using CT/MRI, cystoscopy with biopsy, and urine cytology [50, 52].

Sternberg et al. demonstrated that 72% (*n*= 29) of patients achieving cCR, assessed by CT, TURBT, and urine cytology, and managed with bladder sparing surgery remained alive with a functional bladder at 36 months [39]. Building upon this  Meyer et al. found that among patients who chose bladder preservation after achieving cCR, assessed by CT, cystoscopy with biopsy, and urine cytology, 18 of 25 (72%) retained their bladder at a median follow-up of 55 months [45]. Onishi et al. reported that non-invasive downstaging (cT0/Ta/Tis/T1N0) at TURBT following NAST, assessed by CT and TURBT was associated with 5-year cystectomy-free survival of 85% and overall survival of 89% [40].

Dracham et al. [44] demonstrated that 23% (*n* = 9) and 65% (*n* = 26) of patients achieved complete or major partial response after NAST, assessed by CT and cystoscopy with or without biopsy. At a median follow-up of 43 months, 50% (*n*= 20) retained a functional bladder, with a 3-year bladder-intact disease-free survival rate of 67%. [44]Additional retrospective analyses confirm the association between cCR and sustained bladder preservation:  Yap et al. reported a 53% cCR rate (*n* = 32), assessed by CT and TURBT, with 52% (*n*= 17) maintaining bladder preservation without recurrence within a mean follow-up of 60 months [41]. Herr et al. analyzed cCR patients, assessed by CT and cystoscopy with biopsy, who declined cystectomy, showing 54% bladder preservation at a median follow-up of 86 months [43]. Splinter et al. reported a cCR rate of 7.4% (*n* = 11), assessed by cystoscopy with biopsy. Among patients not undergoing immediate cystectomy, 34% (*n* = 51) received bladder-preserving therapy, with responders to NAST achieving a median survival of 31 months, compared to 18 months in non-responders (*p*< 0.05) [38].

Mofid et al. compared neoadjuvant gemcitabine/cisplatin with gemcitabine/carboplatin followed by chemoradiotherapy and reported comparable cCR rates assessed by CT, cystoscopy with biopsy, and urine cytology [55]. Agrawal et al. observed an increase in cCR rates from 57% after neoadjuvant chemotherapy to 81% following subsequent chemoradiation, assessed by CT and cystoscopy, suggesting an additive effect of sequential multimodal therapy on tumor response [42].

## Discussion

Restaging after NAST in patients with MIBC remains highly inconsistent, with substantial variability in assessment modalities limiting the comparability of cCR outcomes across studies. Among the currently available modalities, MRI demonstrates promising diagnostic accuracy for detecting residual muscle-invasive disease and shows strong correlation with pCR. However, direct comparative evidence between MRI and other assessment modalities remains limited. Current data suggest that sequential multimodal restaging, combining imaging with local clinical evaluation, may offer complementary value and improve the reliability of cCR assessment. Nevertheless, the potential superiority of combined approaches over MRI alone remains hypothetical and requires confirmation in prospective trials.

Several studies have shown limited concordance between clinical and pathological response in MIBC, largely due to inconsistent definitions and varied restaging methods for cCR [57]. While various imaging techniques, local clinical evaluations, and their combinations have been explored as potential restaging tools to predict oncologic outcomes the absence of standardized protocols results in considerable heterogeneity and limits clinical applicability. Consequently, the lack of standardization in current cCR assessment strategies leads to significant discrepancies, limiting their reliability and leaving them as an exploratory approach with considerable uncertainty in guiding bladder-sparing treatment decisions [12]. Notably, current evidence shows that MRI demonstrates favourable diagnostic accuracy and better correlation with pathological response compared to CT as a single imaging modality; however, sequential multimodal restaging provides complementary validation and greater reliability. While most studies focus on primary tumor (pT) response, nodal response may be more prognostically relevant for long-term survival, highlighting the need for accurate assessment of lymph node status.

Against this backdrop of inconsistent and heterogeneous restaging protocols, the advent of urinary tumor DNA (utDNA) and circulating tumor DNA (ctDNA) as novel biomarkers offers a promising and potentially transformative avenue [58–60]. These molecular tools provide an opportunity to fundamentally refine the assessment of cCR, potentially creating a basis for a more precise, biology-driven and standardized definition, especially when combined with mpMRI in a sequential multimodal restaging approach. Recent evidence suggests that utDNA surpasses conventional diagnostic methods by providing high sensitivity and specificity in detecting residual high-grade or muscle-invasive bladder cancer, even at early stages [61]. Its non-invasive character and release from urothelial cells make utDNA well-suited for identifying residual intravesical disease after NAST. In contrast, ctDNA has evolved into a powerful tool for detecting molecular residual disease (MRD) and guiding adjuvant therapy decisions after RC.

In the phase III IMvigor011 trial, among the 250 patients who became ctDNA-positive during post-cystectomy surveillance and were subsequently randomized, adjuvant atezolizumab significantly prolonged disease-free survival (median 9.9 vs. 4.8 months; HR 0.64, 95%CI 0.47–0.87) and overall survival (median 33 vs. 21 months; HR 0.59, 95%CI 0.39–0.90) compared with placebo. Importantly, the 357 patients who remained persistently ctDNA-negative throughout the 1-year surveillance period achieved excellent outcomes, with a 2-year disease-free survival rate of 88% and low recurrence rates in the absence of any adjuvant therapy [62].

The implications of IMvigor011 and similar efforts (NCT04138628) extend directly to the pre-cystectomy setting. Persistent ctDNA positivity despite clinical/imaging-based cCR after neoadjuvant therapy strongly suggests occult systemic micrometastatic disease and may identify patients unlikely to be cured by cystectomy alone, thereby challenging the reliability of current cCR definitions that rely predominantly on imaging [63, 64]. Ongoing and future trials randomizing patients with multimodal cCR (including ctDNA/utDNA negativity) to definitive local therapy vs. bladder preservation will be critical to establish whether such a “response-adapted” approach can safely reduce overtreatment without compromising survival.

The findings of this systematic review should be interpreted considering several limitations. First, the included studies were highly heterogeneous in design, with most being retrospective and involving small patient cohorts, which may introduce selection bias and limit the generalizability of the results. Second, the timing of restaging assessments varied considerably, with some studies performing evaluations immediately before surgery, while others assessed patients after completion of NAST but prior to the final treatment cycle, contributing to variability in response measurement. Third, although cCR was uniformly defined as cT0, the restaging modalities employed differed widely across studies, ranging from local clinical evaluation to imaging, or combinations thereof. Finally, substantial heterogeneity was observed in the neoadjuvant systemic therapy regimens, which included NAST with or without concurrent radiotherapy, further complicating conclusions regarding oncologic outcomes.

## Conclusion

Current cCR assessment strategies in patients with MIBC remain insufficiently accurate and standardized to reliably inform bladder-sparing treatment decisions. Overall, the heterogeneity in cCR assessment methods currently hinders their reliability and clinical applicability, underlining the need for prospective trials directly comparing restaging strategies and integrating novel molecular markers. Importantly, patient outcomes are ultimately driven by treatment decisions, rather than by diagnostic assessments, highlighting the importance of combining accurate restaging with appropriate therapeutic approaches. While combining clinical evaluation and imaging may enhance diagnostic performance, these methods alone cannot ensure consistent cCR determination. Prospective trials integrating utDNA and ctDNA with standardized imaging protocols, such as serial MRI, are needed to establish robust, reproducible frameworks for cCR assessment and to optimize patient selection in bladder-preserving strategies.

## Supplementary Information

Supplementary Material 1.

## Data Availability

No datasets were generated or analysed during the current study.
