# Supplementary material for: Assessing clinical complete response after neoadjuvant systemic therapy in muscle-invasive bladder cancer: a systematic review
Source: World J Urol. 2025 Dec 24;44(1):65. doi: 10.1007/s00345-025-06146-7 (PMC12738659; doi:10.1007/s00345-025-06146-7)

**Supplementary File 1: Preferred Reporting Items for Systematic Reviews and Meta-analyses (PRISMA) - flow diagram for new systematic reviews which included searches of databases and registers only**

**Report included (n = 41)**

- cCR treatment decisional (n=22)
- cCR not treatment decisional (n=19)

**Identification of studies via databases and registers**

**Screening**

Records screened

(n=778)

Records excluded

(n=544)

Reports sought for retrieval

(n=234)

Reports not retrieved

(n=0)

Reports assessed for eligibility; Full-text screening

(n=234)

Reports excluded

- cCR after NAST not assessed (n=169)
- RT with NAST prior cCR (n=14)
- no separation between UTUC and BCa

(n=4)

- mBCa/advanced BCa (n=5)
- non-conventional histology; recurrence of UTUC (n=2)

Reports included (backwards citation)

- n=1

**Included**

**Identification**

Records identified by database searching n=2362

MEDLINE: n=654

Embase: n=1000

Web of Science: n=708

Reports excluded

- Review (n=330)
- Case-Report

(n=56)

- Editorial/Comment/Conference Abstract

(n=458)

- non-English (n=74)
- Duplicates (n=666)

Source: Page MJ, et al. BMJ 2021;372:n71. doi: 10.1136/bmj.n71.

This work is licensed under CC BY 4.0. To view a copy of this license, visit <https://creativecommons.org/licenses/by/4.0/>

**Supplementary File 2: Detailed Search Strategy for the databases**

**MEDLINE (PubMed) – 25/07**

| 1 | (  "bladder cancer*"[Title/Abstract]  OR "urothelial carcinoma*"[Title/Abstract]  OR "urothelial cancer*"[Title/Abstract]  OR "bladder carcinoma*"[Title/Abstract]  OR "Urinary Bladder Neoplasms"[MeSH Terms]  OR "Carcinoma, Transitional Cell"[MeSH Terms]  ) | 94299 |
| --- | --- | --- |
| 2 | (  "neoadjuvant"[Title/Abstract]  OR "preoperative"[Title/Abstract]  OR "perioperative"[Title/Abstract]  OR "Neoadjuvant Therapy"[MeSH Terms]  OR "NAC"[Title/Abstract]  OR ("neoadjuvant"[Title/Abstract] AND "immunotherapy"[Title/Abstract])  ) | 583016 |
| 3 | (  "restaging"[Title/Abstract]  OR "tumor response"[Title/Abstract]  OR "response assessment"[Title/Abstract]  OR "treatment response"[Title/Abstract]  OR "clinical response"[Title/Abstract]  OR "clinical complete response"[Title/Abstract]  OR "cCR"[Title/Abstract]  OR "complete response"[Title/Abstract]  OR "cT0"[Title/Abstract]  ) | 152265 |
|  | 1 AND 2 AND 3 | 654 |

**Embase – 25/07**

| 1 | (  'urinary bladder neoplasm'/exp  OR 'bladder cancer'/exp  OR 'urothelial carcinoma'/exp  OR 'transitional cell carcinoma'/exp  ) | 149721 |
| --- | --- | --- |
| 2 | (  'neoadjuvant therapy'/exp  OR 'preoperative care'/exp  OR 'perioperative period'/exp  OR 'neoadjuvant immunotherapy'/exp  ) | 1407183 |
| 3 | (  'restaging'/exp  OR 'tumor response'/exp  OR 'treatment response'/exp  OR 'response evaluation'/exp  OR 'clinical response'/exp  OR 'complete clinical response'/exp  OR 'complete remission'/exp  ) | 433671 |
|  | 1 AND 2 AND 3 | 1000 |

**Web of Science – 25/07**

| 1 | TS=(  "bladder cancer*"  OR "urothelial carcinoma*"  OR "urothelial cancer*"  OR "bladder carcinoma*"  OR "urinary bladder neoplasm*"  OR "transitional cell carcinoma*"  )  AND  TS=(  "neoadjuvant"  OR "preoperative"  OR "perioperative"  OR "neoadjuvant therapy"  OR "NAC"  OR ("neoadjuvant" AND "immunotherapy")  )  AND  TS=(  "restaging"  OR "tumor response"  OR "response assessment"  OR "treatment response"  OR "clinical response"  OR "clinical complete response"  OR "cCR"  OR "complete response"  OR "cT0"  ) | 708 |
| --- | --- | --- |


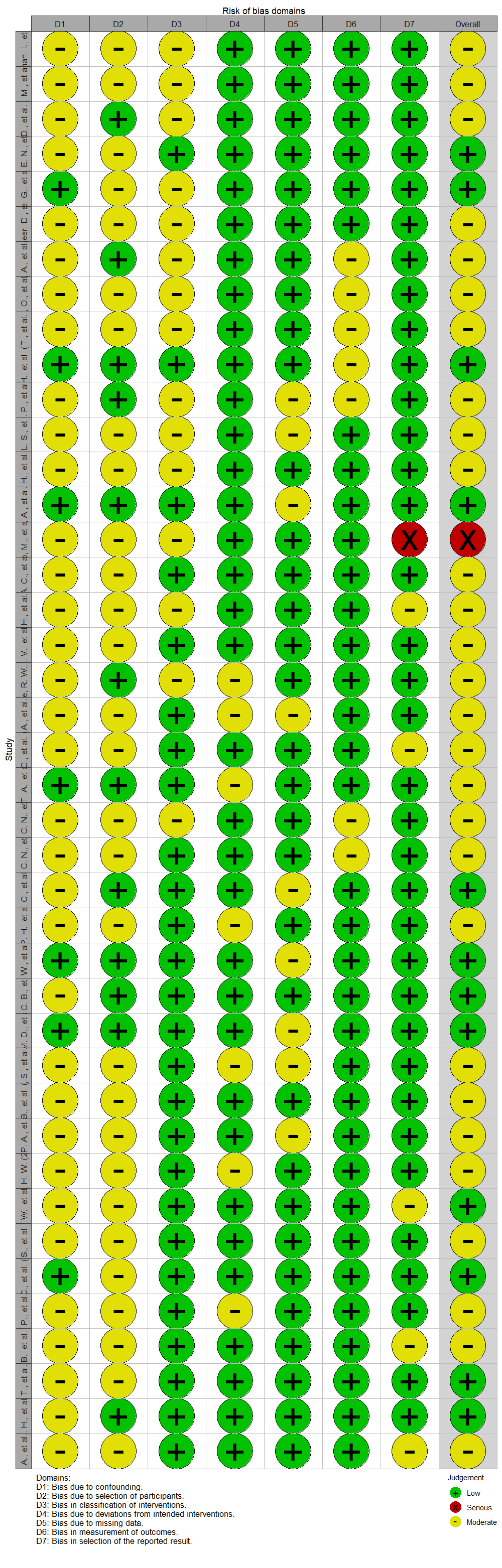
**Supplementary File 3: Risk of Bias according to ROBINS-I tool**

**Supplementary File 4: PEO Framework**

P:

Adults (≥18 years) diagnosed with muscle-invasive bladder cancer (MIBC)

Patients who have received neoadjuvant systemic therapy

E:

Assessment of clinical complete response (cCR, pT0) through cystoscopy, imaging modalities (e.g., CT, MRI), and/or urine cytology following neoadjuvant systemic therapy.

O:

Clinical complete response (cCR) assessment modalities with or without their association with pathological complete response (pCR), including sensitivity, specificity, positive predictive value (PPV), and negative predictive value (NPV) and/or overall oncologic outcomes (e.g. metastatic free survival, overall survival)

S:

Prospective and retrospective reports, randomized-controlled trials, post-hoc and subsequent analyses of trials

**Supplementary File 5: AMSTAR 2 Checklist**


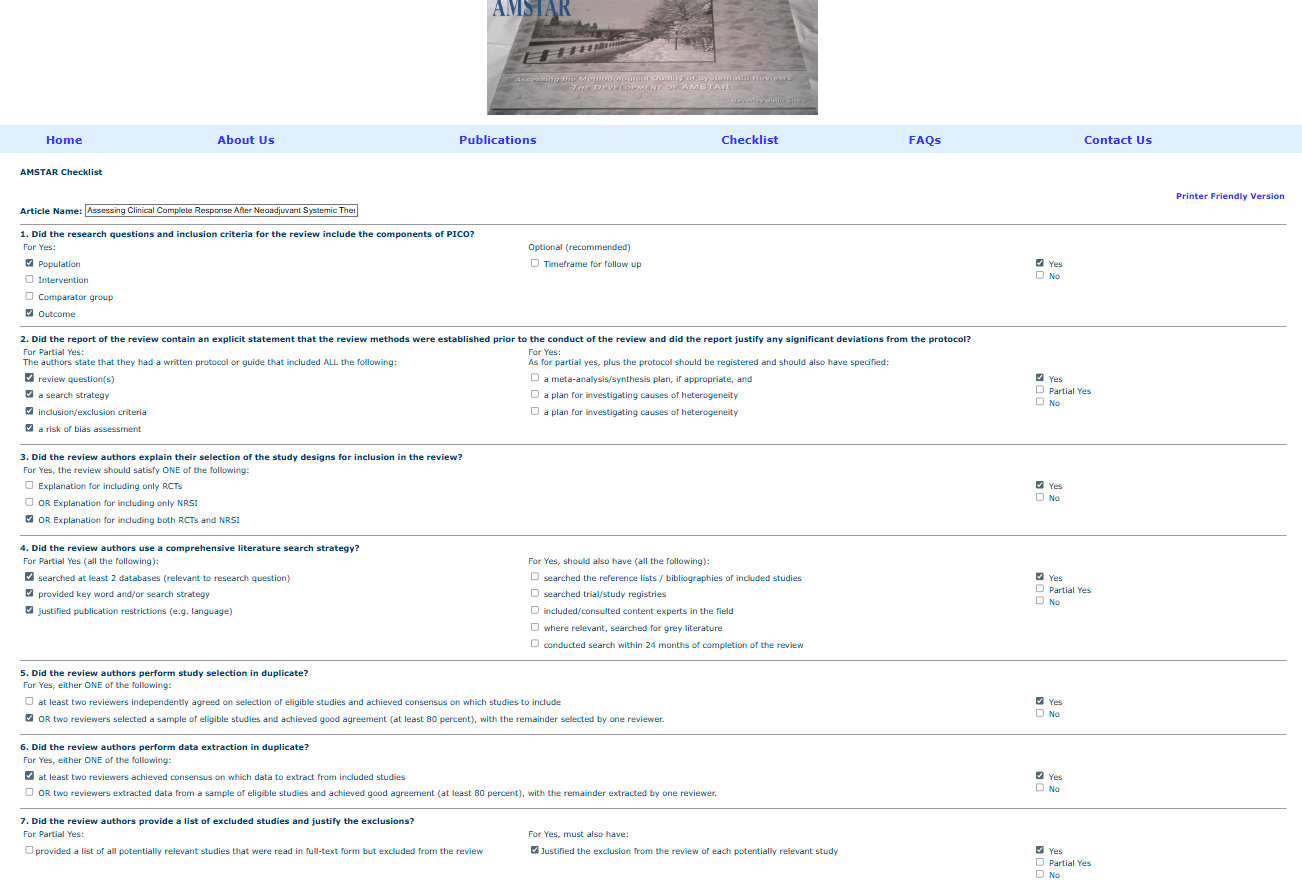

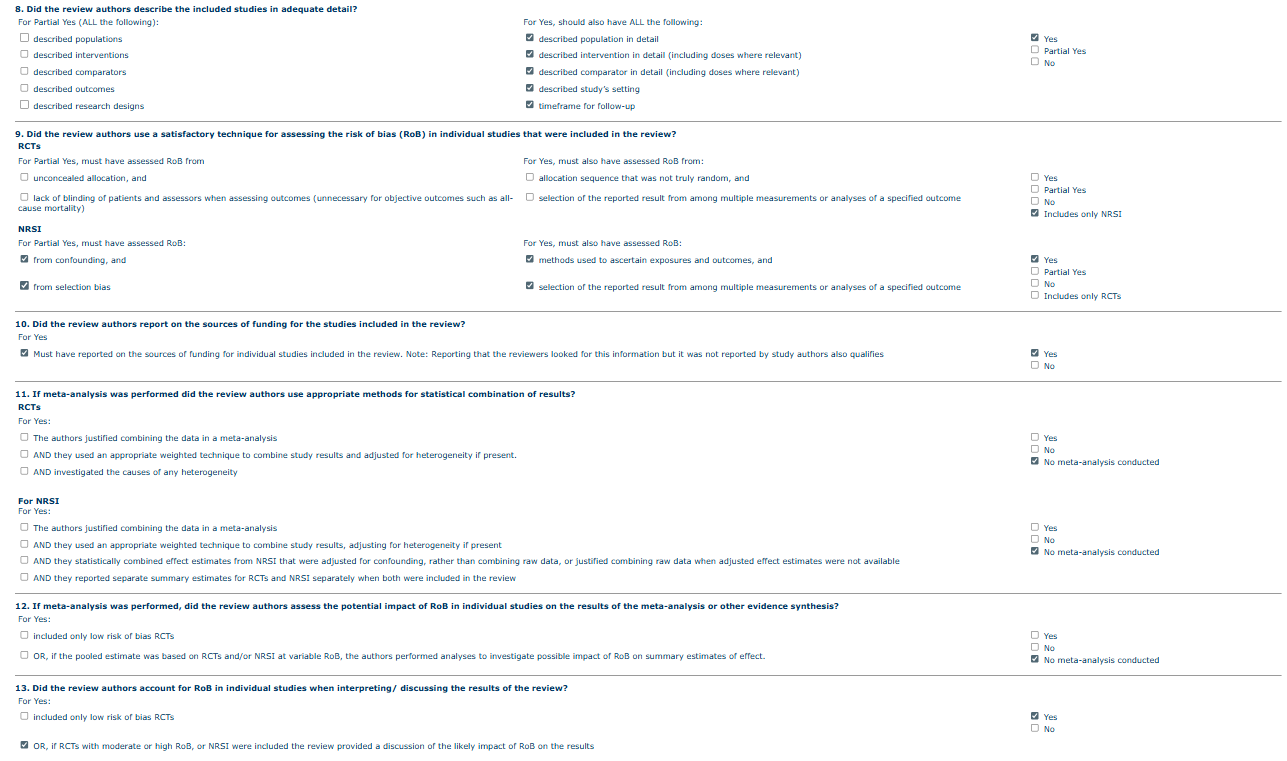

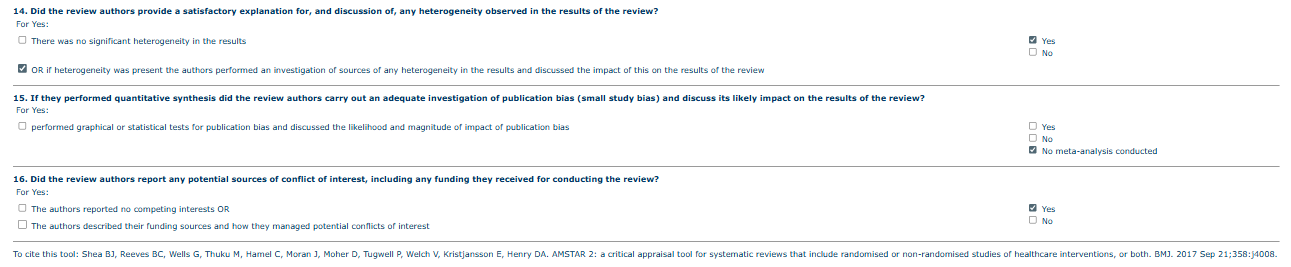

Supplement: Supplementary file 1 — Supplementary Material 1. [file 345_2025_6146_MOESM1_ESM.docx]
